# Low-Frequency Oscillatory Correlates of Auditory Predictive Processing in Cortical-Subcortical Networks: A MEG-Study

**DOI:** 10.1038/s41598-018-32385-3

**Published:** 2018-09-18

**Authors:** Marc Recasens, Joachim Gross, Peter J. Uhlhaas

**Affiliations:** 1 Institute of Neuroscience and Psychology, University of Glasgow, 58 Hillhead Street, Glasgow, G12 8QB Scotland United Kingdom; 2 Institute of Biomagnetism and Biosignalanalysis, University of Muenster, Malmedyweg 15, 48149 Muenster, Germany

## Abstract

Emerging evidence supports the role of neural oscillations as a mechanism for predictive information processing across large-scale networks. However, the oscillatory signatures underlying auditory mismatch detection and information flow between brain regions remain unclear. To address this issue, we examined the contribution of oscillatory activity at theta/alpha-bands (4–8/8–13 Hz) and assessed directed connectivity in magnetoencephalographic data while 17 human participants were presented with sound sequences containing predictable repetitions and order manipulations that elicited prediction-error responses. We characterized the spectro-temporal properties of neural generators using a minimum-norm approach and assessed directed connectivity using Granger Causality analysis. Mismatching sequences elicited increased theta power and phase-locking in auditory, hippocampal and prefrontal cortices, suggesting that theta-band oscillations underlie prediction-error generation in cortical-subcortical networks. Furthermore, enhanced feedforward theta/alpha-band connectivity was observed in auditory-prefrontal networks during mismatching sequences, while increased feedback connectivity in the alpha-band was observed between hippocampus and auditory regions during predictable sounds. Our findings highlight the involvement of hippocampal theta/alpha-band oscillations towards auditory prediction-error generation and suggest a spectral dissociation between inter-areal feedforward vs. feedback signalling, thus providing novel insights into the oscillatory mechanisms underlying auditory predictive processing.

## Introduction

The ability to extract statistical regularities from the acoustic scene is a fundamental goal for cognitive systems as it serves as the basis for predictive modelling and detection of environmental changes^1^. Signatures of mismatch detection in the human auditory system have been traditionally linked to event-related potentials (ERP), such as the Mismatch Negativity (MMN) or the P300 complex^2–5^. Within a predictive coding framework, mismatch and novelty-related ERP signatures are interpreted as prediction-error responses that signal the mismatch between sensory inputs and internal models, thus leading to an on-line process of prediction comparison and subsequent model refinement^6–8^.

The majority of research in auditory mismatch detection has focused on ERP signatures^9,10^. However, less attention has been paid to the contribution of rhythmic activity towards the detection of matching and mismatching auditory events. Neural oscillations play an important role in routing information within and across brain regions^11,12^ as well as controlling information gating and maintaining sensory representations^13–16^. In addition, increasing evidence indicates that neural oscillations are fundamental for the signalling of top-down predictions and bottom-up prediction-errors conveyed across hierarchical regions in distinct oscillatory bands^12,17–19^. Specifically, theta- and gamma-band oscillations appear to convey feedforward-mediated prediction-errors, whereas alpha- and beta-band rhythms are predominantly involved in the mediation of top-down feedback signals^12,17,19–21^.

Previous studies have indicated the role of low-frequency (theta 4–8 Hz, and alpha 8–13 Hz) oscillations within a frontal-temporal-parietal network underlying MMN and P300 processes^4,22–27^. While preliminary evidence suggests the involvement of theta-band oscillations during novelty processing^28^, the possible contribution of neural oscillations towards prediction-error generation during auditory mismatch detection remains unclear. One possibility is that the hippocampus plays a prominent role in auditory mismatch detection as indicated by evidence from ERPs, such as the P300^29–31^. In addition, hippocampal theta-band modulation has been observed in response to both environmental novelty^32,33^ and prediction-mismatching events^34,35^ in the visual domain.

**Figure 1:**
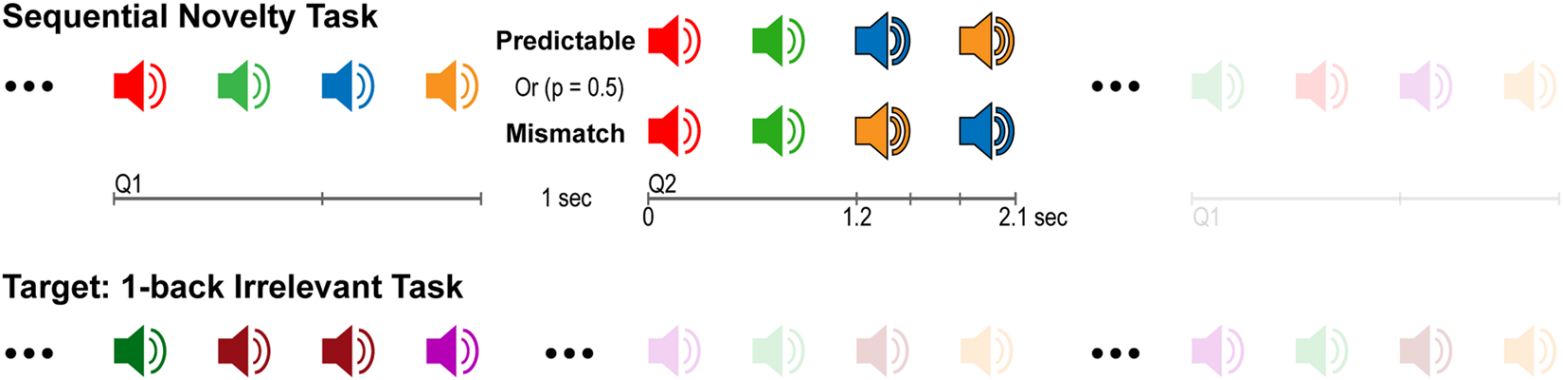
Experimental design. Serial presentation of 4-sounds sequences spaced by 1 s intervals (upper plot). Fifteen possible frequencies (100 to 800 Hz, plus 1st and 2nd harmonics, 10 ms rise/fall, linearly spaced in 50 Hz steps) leading to 32760 different possible frequency-order combinations. Participants were instructed to perform an irrelevant 1-back task (lower plot) and detect rare sequences containing a sound repetition (two sounds presented in a row within the sequences).

In the current study, we addressed these questions through the acquisition of MEG-data from healthy participants during the presentation of sound sequences that were followed by either a repetition or by a sequence containing a manipulation in the order of the last two sounds (Fig. 1). As opposed to novelty processing, our aim was to elucidate comparison processes whereby inputs match or mismatch implicit predictions. Therefore we did not include an unpredictable condition as in previous studies^34–37^. Unlike conventional oddball designs where the magnitude of hippocampal (HP) novelty responses decrease over the course of the experiment^38,39^, we presented four-sound sequences (quartets) while participants performed an orthogonal auditory task. After a short delay, a second quartet was presented, either in exactly the same order as the previously presented sequence, or with the last two sounds presented in reverse order. The first two sounds were always identical to those in the previous quartet, thus in both cases predictable and unpredictable processes were contingent on a prior matching process. Moreover, the sequential nature of predictions and mismatch responses in such a design maximized the recruitment of HP-prefrontal cortex (HP-PFC) circuits^34–36,40–42^.

Consistent with a predictive coding framework^17^, we hypothesized that mismatching auditory events would elicit a prediction-error response reflected by increased theta-band activity and feedforward information-flow from sensory to PFC regions, while matching or predictable sequence**s** would be characterized by an attenuated prediction-error response as well as increased alpha-band feedback signalling from regions involved in the encoding sequential regularities, such as the HP and PFC.

## Results

**Figure 2:**
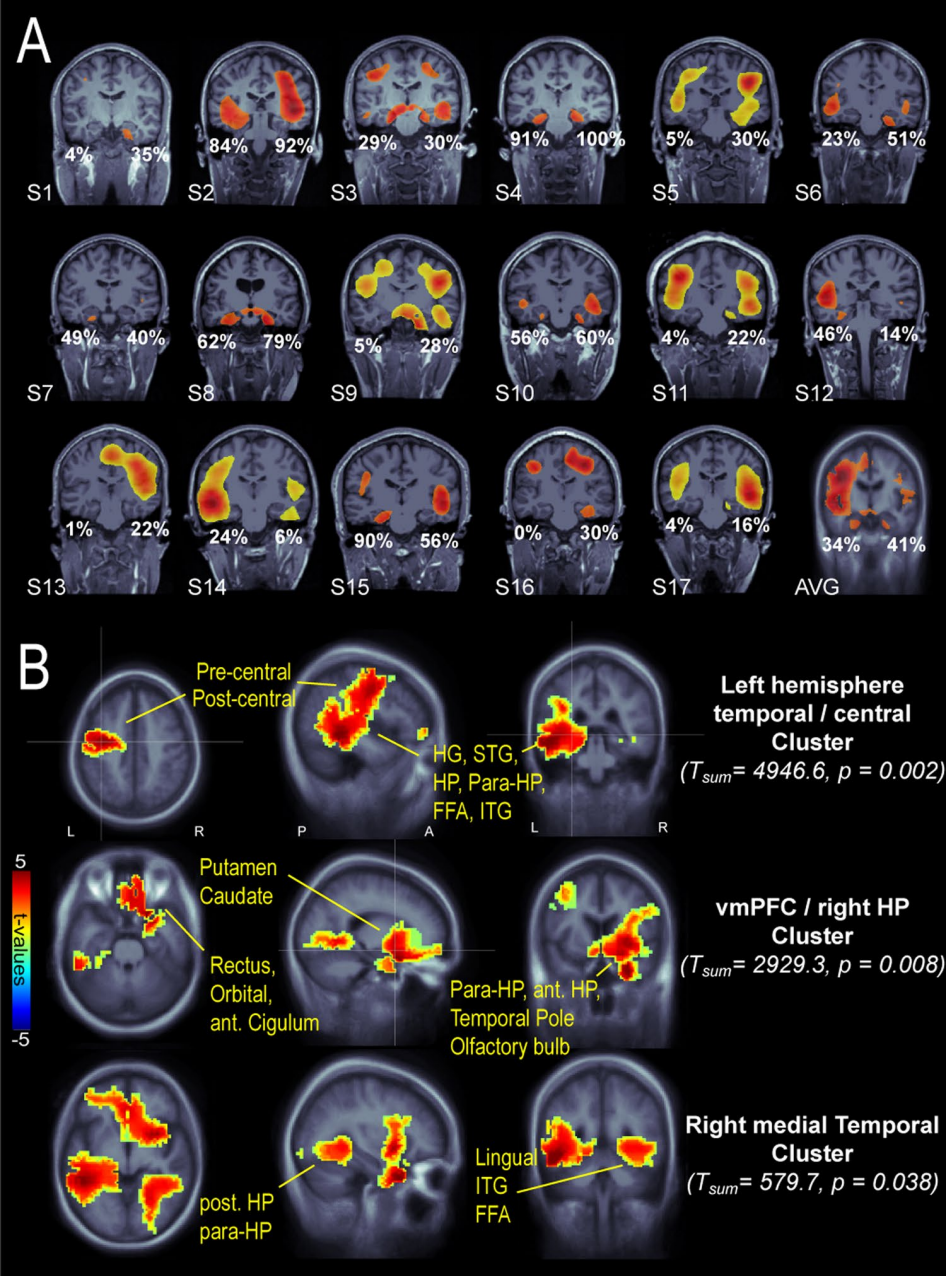
Whole-brain evoked-theta effects during the post-violation interval (1.2–2.1 s). (**A**) Individual coronal slices showing hippocampal structures. Evoked theta dSPM activity is thresholded at 75% of maximal activation, except for subjects S5, S9, S11, S13, S14, S17 (60% threshold) and subject average (AVG, 50% threshold). Percentages indicate the fraction of voxels in left and right HP exceeding the 75% of maximal activation. (**B**) Group-level statistics: Three clusters show significantly larger activity for mismatch vs predictable condition (P < 0.05, corrected for multiple comparisons across neighbouring spatial bins). Abbreviations: Post/ant-HP (posterior/anterior hippocampus), para-HP (para-hippocampal), dlPFC (dorsolateral prefrontal cortex), vmPFC (ventromedial prefrontal cortex), HG (Heschl’s gyrus), STG (superior temporal gyrus), FFA (fusiform area), ITG (inferior temporal gyrus).

We first identified individual brain areas involved in auditory match and mismatch detection by filtering sensor-level trials in the theta band (4–8 Hz) and computing the difference between “mismatch” vs. “predictable” averaged data. Whole-brain evoked theta-power was estimated using a noise-normalized minimum-norm estimate (MNE) known as dynamic statistical parametrical mapping (dSPM). Individual dSPM maps (Fig. 2A) showed that theta-band activity in medial temporal lobe (MTL) and vmPFC could be reliably detected in individual participants (MTL: n = 15; vmPFC: n = 9; at a threshold of 60%). Across participants, 42% of the grid points in the left HP and 34% in the right HP exceeded the 75% of the maximal whole-brain dSPM activation, supporting a likely engagement of hippocampal sources. At the group-level, cluster-corrected non-parametric statistics were applied for dSPM volumetric maps in the post-violation interval (1.2–2.1 s), revealing regions where evoked theta-activity was enhanced for mismatch as compared to predictable sequences (p < 0.05; cluster-corrected at p < 0.001) in the left MTL and left central areas, vmPFC and right anterior temporal lobe, and right posterior MTL (Fig. 2B). These results so far demonstrate that the presentation of acoustic order-violations elicits increased theta-band activity in a large-scale network involving cortical and hippocampal structures.

### Low-Frequency Oscillations in HP-PFC Circuits Underlie Auditory Mismatch Detection

**Figure 3:**
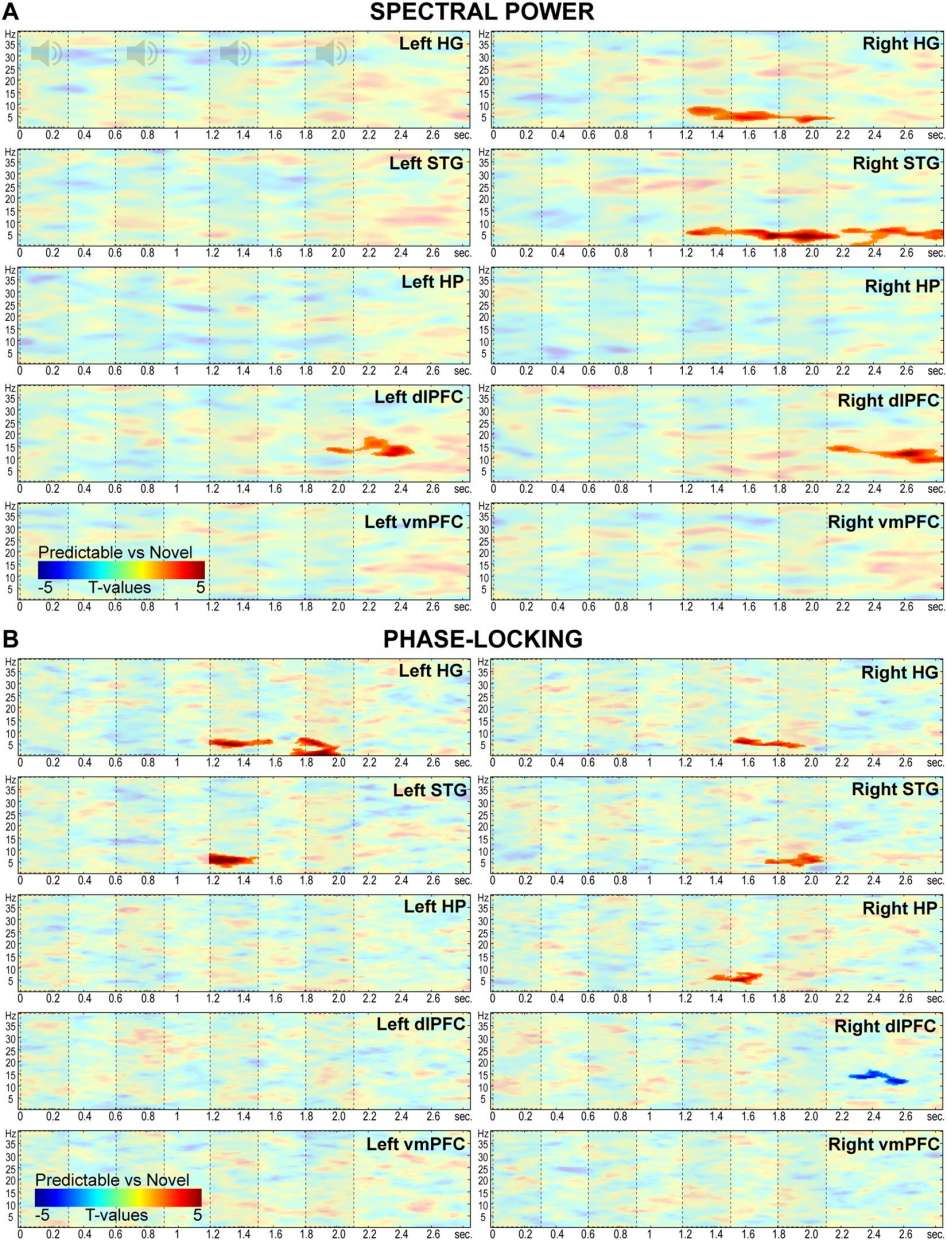
Oscillatory analyses in HG, STG, HP, dlPFC and vmPFC. Fully saturated colors highlight clusters of significant modulation between 1.2 and 2.1 s (Red: mismatch > predictable; Blue: predictable > mismatch; P < 0.05, two-sided, corrected for multiple comparisons across time-frequency bins). Dashed boxes indicate onset-offset of sounds in the sequence. (**A**) Spectral power contrasts (mismatch vs predictable). (**B**) Phase-locking (inter-trial phase coherence) contrasts (mismatch vs predictable).

We computed time-frequency response (TFR) maps of spectral power and phase-locking from cortically-constrained trial-level MNE-estimates between 1 and 40 Hz and from 0 to 2.9 s in each ROI. Condition differences were statistically contrasted within the post-violation interval using non-parametric dependent-sample t-tests and clustering for time-frequency bins (Fig. 3). Mismatch sequences elicited enhanced 2–9 Hz power in right HG and STG compared to predictable sequences (HG: 1.21–2.15 s; T_sum_ = 1410.7; p = 0.003; STG: 1.2–2.18 s; T_sum_ = 1927.1; p = 0.0035). A second cluster in the right STG showed increased theta-band power after auditory stimulation (2.2–2.8 s; T_sum_ = 1083.7; p = 0.0225) as well as increased phase-locked activity in the 3–8 Hz range in the right HG (1.5–1.9 s; T_sum_ = 562.6; p = 0.018) and right STG (1.7–2.1 s; T_sum_ = 589.6, p = 0.021). Similar phase-locked effects were observed in the left hemisphere (left HG: 1.2–1.6 s, T_sum_ = 648; p = 0.013; and 1.7–2 s; T_sum_ = 968.2; p = 0.001; left STG 1.2–1.5 s; T_sum_ = 892.2; p = 0.002). Notably, following the modulation of theta-band oscillations in auditory areas, the right HP showed increased mismatch-related phase-locked activity in the 3–8 Hz range between 1.3–1.7 s (T_sum_ = 562.6, p = 0.018). In addition to theta-band modulation, enhanced power in the alpha/beta-band for mismatch as compared to predictable sequences was observed in the right dlPFC (~7–16 Hz; 2.1–2.8 s; T_sum_ = 1329.8; p = 0.008) and left dlPFC (~11–19 Hz; 1.9–2.5 s; T_sum_ = 1042.6; p = 0.02). In contrast, one cluster in the right dlPFC showed increased phase-locked activity for predictable as compared to mismatch trials in the alpha-band (~10–16 Hz; 2.2–2.6 s; T_sum_ = −550.6; p = 0.013). These results confirmed that mismatch sequences elicited enhanced theta-band activity in auditory and HP regions during the presentation of order violations, thus highlighting the role of theta-rhythms and HP sources in the generation of prediction-error signals.

### Mismatch Negativity in auditory-HP-PFC circuits

**Figure 4:**
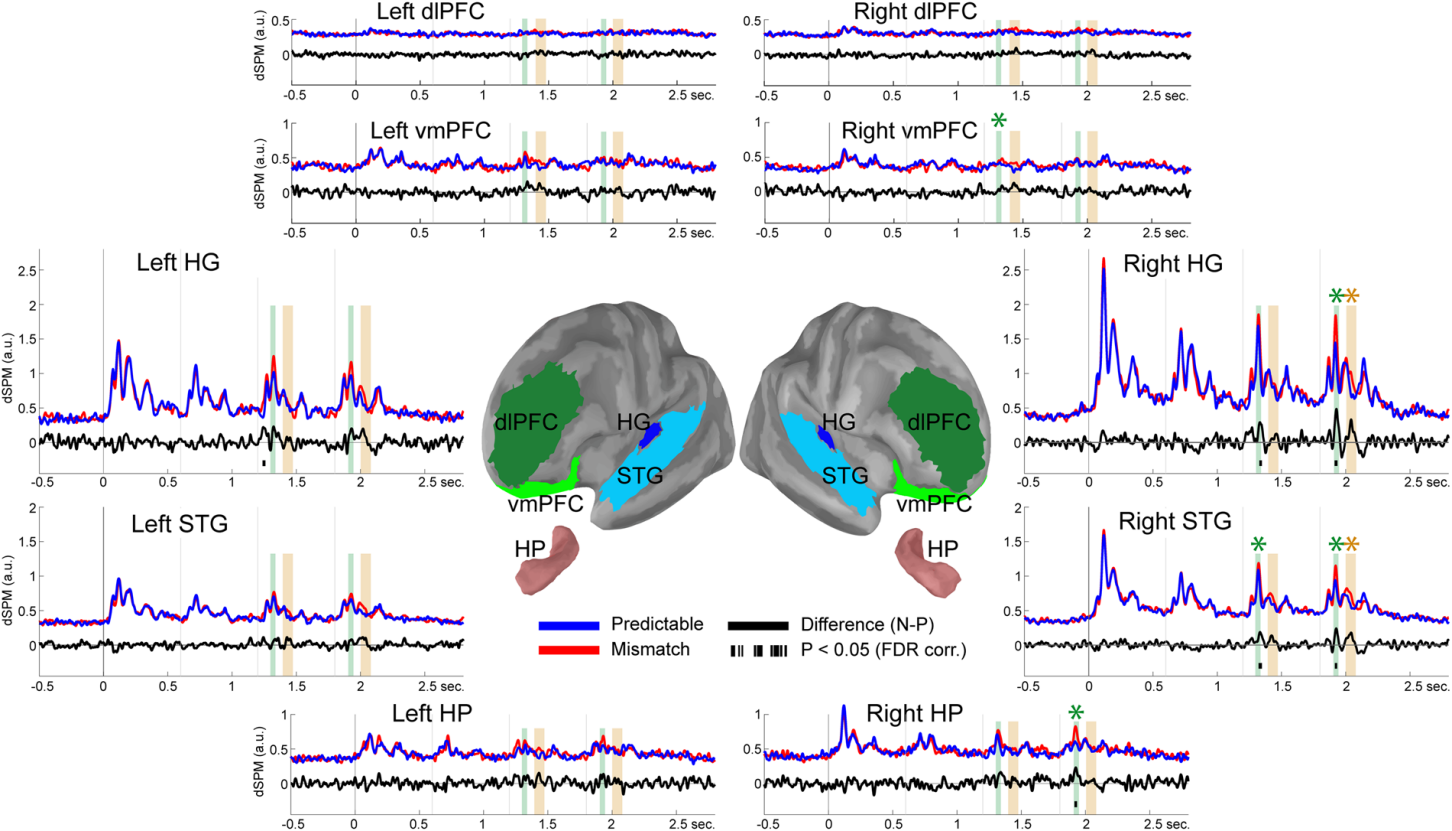
Evoked-responses in HG, STG, HP, dlPFC and vmPFC. Central panel depicts ROIs overlaid onto an inflated template cortical surface. Surrounding panels illustrate group-averaged dSPM time-courses in each bilateral ROI for mismatch (red lines), predictable (blue lines) and difference (mismatch minus predictable; black lines) conditions. Black bars below the zero line indicate clusters showing significant differences between conditions (P < 0.05 two-sided, FDR corrected across ROIs). Vertical bars indicate the MMN (green bars: 1.3–1.34 s/1.9–1.94 s) and P300 (yellow bars: 1.4–1.48 s/2–2.08 s) time-intervals used for statistical comparison. Asterisks indicate statistically significant differences (P < 0.05 two-sided, FDR corrected across ROIs) between mismatch and predictable conditions in the MMN and P300 intervals.

To relate oscillatory findings to the extensive literature on ERP/ERFs linked to deviance detection, we analysed broadband filtered (1–30 Hz) average time-series in each ROI (Fig. 4). Exploratory analyses conducted across time-points (0 to 2.8 s) revealed significant differences across conditions as early as ~50 ms after the onset of a mismatch sound in the left HG (1.238–1.259 s; t(16) = 3.57; p = 0.0026). Subsequent effects were observed ~120 ms after the onset of the third and fourth sounds in the right HG (1.328–1.348 s: t(16) = 2.95, p = 0.0092; 1.914–1.936 s: t(16) = 4.06, p = 0.002), right STG (1.322–1.348 s: t(16) = 3.53, p = 0.0021; 1.918–1.934 s: t(16) = 3.86, p = 0.0029) and right HP (1.914–1.934 s; t(16) = 3.11; p = 0.0099). Post-hoc analyses confirmed that compared to predictable sounds, mismatch stimuli presented in the third position elicited an enhanced response in the MMN time range (1.300–1.340 ms) in the right STG (t(16) = 3.88, p = 0.0007) and vmPFC (t(16) = 3.61, p = 0.0035). Similarly, **a** MMN response was observed in response to the fourth stimulus (1.900–1.940 ms) in the right HG (t(16) = 4.16, p = 0.0013), STG (t(16) = 3.68, p = 0.0021), and HP (t(16) = 3.31, p = 0.0065). Finally, an enhanced response was observed at ~200 ms after the onset of the fourth sound (2.000–2.080 ms) in the right HG (t(16) = 3.21, p = 0.0037) and STG (t(16) = 3.33, p = 0.0003).

### Theta- and Alpha-Band Oscillations Mediate Transmission of Feedforward and Feedback Predictive Signals

**Figure 5:**
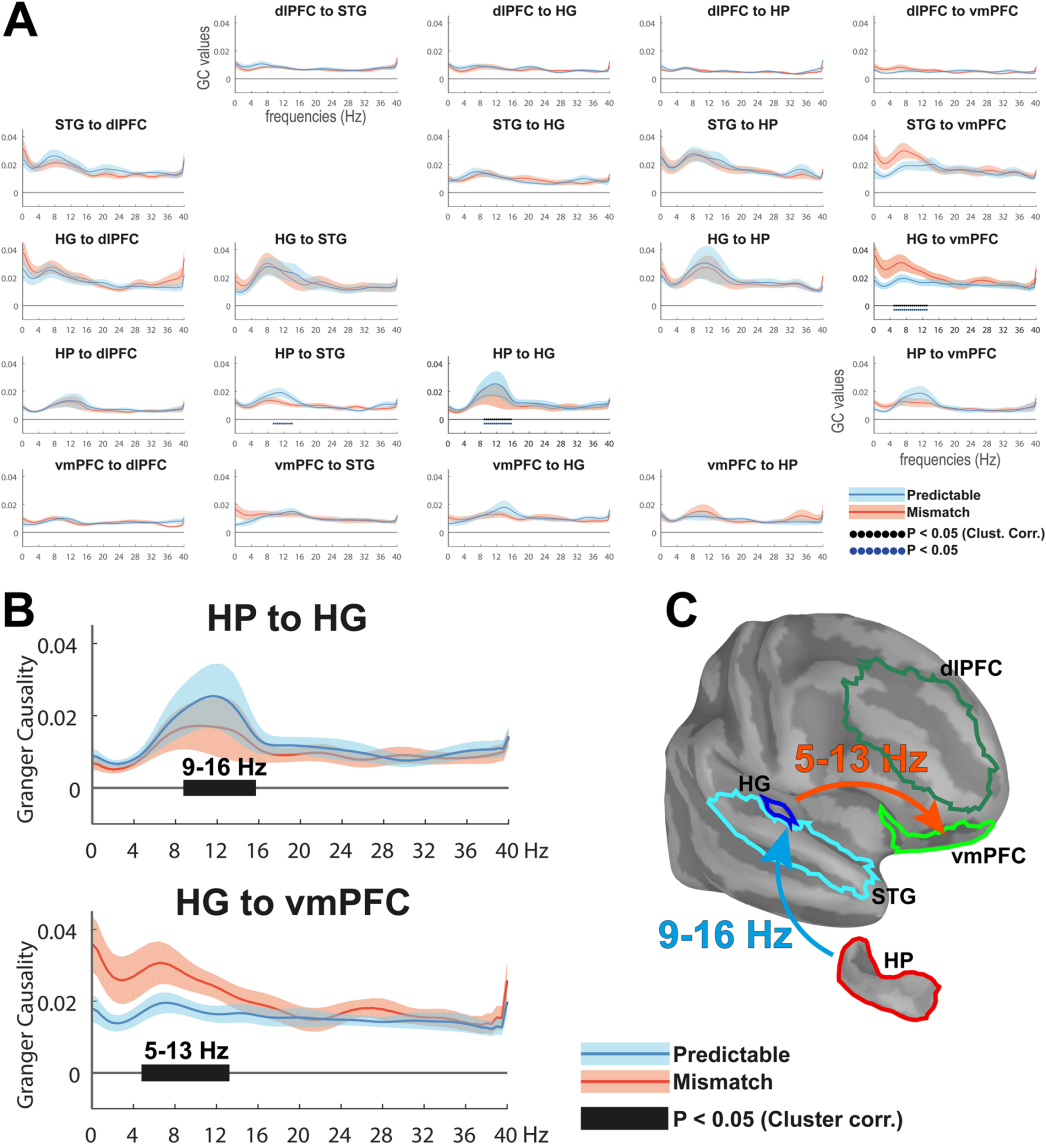
Spectrally resolved Granger Causality (GC) analysis. (**A**) GC-estimates for mismatch (red lines) and predictable (blue lines) conditions across all pairs of anatomical regions in the right hemisphere (Shaded areas around lines indicate standard error. Black dotted lines indicate clusters showing significant differences between conditions (P < 0.05 two-sided, corrected for multiple comparisons across frequency bins. Blue dotted lines indicate uncorrected clusters). (**B**) Zoomed-up insert of the two pairs of nodes showing GC differences across conditions in the theta- and alpha-bands. (**C**) Schematic summary of directed GC-results.

We applied frequency-domain multivariate GC to examine directed connectivity patterns during the processing of mismatch and predictable sequences across ROIs in each hemisphere (Fig. 5). GC is a statistical measure that quantifies the extent to which activity in one region predicts activity in another region and has been previously used to identify feedforward and feedback information flow^12,20,43^. GC was measured across all pairs of ROIs per hemisphere in the 1–40 Hz frequency range by segmenting the post-violation interval into 3 non-overlapping 300 ms bins. A non-parametric statistical contrast was applied to assess directed connectivity patterns during mismatch vs predictable sequences. Consistent with our hypothesis that theta/alpha-band oscillations mediate the transmission of prediction-error signals across cortical-subcortical networks, we observed significantly enhanced GC in the 5–13 Hz ranges from right HG to right vmPFC during mismatch as compared to predictable sequences (T_sum_ = 47.4; p = 0.011). In contrast, elevated GC-connectivity from the right HP to the right HG at alpha-frequencies (9–16 Hz; T_sum_ = −45.8; p = 0.006) indicated increased information transfer during predictable vs. mismatch quartets. No statistically significant differences were observed across left hemispheric ROIs. These results indicate that prediction-error signals are projected in a bottom-up fashion from sensory to high-order areas in the PFC, whereas predictable sequences elicit a top-down modulation from HP to auditory cortices. Moreover, our results suggest the use of distinct but overlapping frequency channels in the transmission of feedforward and feedback predictive signals. Analyses aimed at assessing the confounding effects of linear noise mixing showed no significant differences between conditions. Likewise, none of the surrogate time-reversed analyses replicated the enhanced theta/alpha GC from right HG to right vmPFC during mismatch events, or the enhanced alpha GC from the right HP to both right HG and STG during predictable sounds. Overall, these findings suggest that connectivity was robust and could not be accounted for by differences in noise levels across ROIs or the detrimental effect of evoked activity on GC estimation.

## Discussion

We used MEG to identify oscillatory and connectivity signatures of match and mismatch detection. While the functional role of neural oscillations in visual processing has gained considerable support^12,19,20,44,45^, the relevance of rhythmic activity and in particular the specific frequency channels that underlie information processing during auditory perception are less clear. We report that auditory events containing sequential violations with respect to previously presented sequences elicit increased theta-band activity in auditory and HP cortices and modulate PFC alpha-band activity. Increased theta- and alpha-band activity in auditory-HP-PFC circuits was accompanied by novelty-related evoked responses such as the MMN, suggesting that the HP is involved in mismatch detection. Furthermore, theta- and alpha-band connectivity across auditory-HP-PFC regions was modulated by predictable vs unpredictable sound sequences, suggesting that low-frequency oscillations mediate directed information flow across cortical-subcortical networks during auditory predictive processing.

Specifically, we identified increased activity in an auditory-HP-PFC network during auditory mismatch detection that overlapped with neural generators underlying novelty-related ERPs such as auditory, hippocampal, and prefrontal regions^2,4^. Spectral analysis indicated increased power and phase-locking of theta-band oscillations in auditory regions, suggesting that theta oscillations are involved in the generation of early signatures of auditory prediction-error. In contrast, oscillatory activity in bilateral dlPFC was characterized by a late enhancement of alpha power during mismatch trials, concurrent with increased phase-locked activity in the right dlPFC for predictable sequences. Previous studies have reported a correlation between auditory novelty and alpha power at frontal sites reflecting attentional engagement^4,46^. Conversely, increased phase-resetting in dlPFC during the completion of sound quartets is consistent with the involvement of recall processes driven by the match between predictable sequences and identical memory traces^47,48^.

A key aspect of our finding was the observation of enhanced theta phase-resetting in the right HP that followed activity in primary auditory areas. Previous findings have described HP generators underlying the P300 response^29,31,49^ as well as the involvement of HP theta-band activity in the detection of novel events in the visual domain^32–35,50^. Our findings show that HP theta phase-alignment is increased during auditory mismatch detection, thus adding to growing evidence that the HP-entorhinal system sustains a domain-general mechanism encoding sequential representations across spatial and non-spatial dimensions and sensory modalities^40,51–53^. Furthermore, the reliable localization of theta-band modulation in individual participants in HP supports the use of MEG to detect source activity in limbic regions, known to be challenging due to the decay of the magnetic fields with increasing distance from sensors^35,54–59^.

Time-Frequency analyses showed that mismatch-related modulation was band-limited to the alpha/theta range and showed significant phase-locked components in auditory and HP regions during the post-violation interval. Maximal phase-resetting occurred in auditory regions within the 300 ms duration of mismatch sounds, consistent with broad-band evoked responses showing enhanced ERP-components to mismatch sounds in right hemispheric auditory-HP-PFC circuits. This finding suggests that theta-modulation elicited by mismatching stimuli is in part reflected by transient ERP components. The fact that only mismatching sounds at the end of the sequence elicited a late enhanced response around ~200 ms suggests that P2 components might index the encoding of sequence-order violations. Consistent with prior findings, our spectral data suggest that phase-resetting in the HP might reflect the emergence of late hippocampal P300 subcomponents^31,32,49^. Noteworthy, ERF-analysis showed that HP-activity was enhanced at ~120 ms after the onset of the second mismatch sound during the time-range of the MMN potential, thus suggesting that HP generators are involved not only during auditory target detection^29^ but also in early stages of automatic mismatch processing.

While our findings are in agreement with Fuentemilla and colleagues^25^ indicating that MMN generation in auditory sites correlates with phase-alignment of the theta rhythm, our results suggest that auditory mismatch detection correlates with an overall increase in total theta/alpha power even beyond the time-intervals of the MMN and P300 components in the right STG and bilateral dlPFC^22,23^. Previous studies have shown that theta-band oscillations are involved in the maintenance and updating of auditory memory contents, while increased alpha activity in PFC is consistent with an increased memory or suppression of irrelevant processing as observed in short-term visual and auditory memory studies^60,61^. The involvement of theta oscillations in frontal and parietal sites during sequential working memory processes is well-documented^53^, which suggests that memory-based mechanisms could be involved in the current study. However, the relatively early onset of auditory responses in both evoked and spectral analyses suggests that associative mismatch processes between sensory predictions and sensory inputs occurred at a pre-attentive stage. For instance, neither matching nor mismatching events elicited a modulation of the P300 component, which has been typically associated to active monitoring and target detection^4,5^. Moreover participants were actively engaged in a 1-back detection task that oriented their attention to the tones within each quartet. For these reasons, it is unlikely that participants were actively creating explicit predictions about the forthcoming sequences.

Analyses of spectral connectivity allowed us to identify the role of low-frequency oscillations during the processing of mismatch vs predictable events. Information flow from auditory cortices to vmPFC between 5–13 Hz was observed for mismatch sequences, while connectivity from the HP to auditory regions at alpha-frequencies was increased for predictable sounds. Together with the findings of phase-resetting and modulation of theta/alpha-band power in auditory-PFC-HP circuits, these data suggest that auditory mismatch detection involves a coordinated sequence of oscillatory processes whereby prediction-errors are initially generated in sensory regions, followed by secondary auditory and HP cortices and then propagated to vmPFC. Such bottom-up message passing is consistent with studies pointing towards a critical role of PFC in the generation and updating of online predictions and schema-based memory formation^40,41,52,62,63^. In contrast, elevated top-down connectivity from HP to HG in the alpha-band was observed during predictable sequences. This dissociation between theta vs. alpha-band activity is consistent with current frameworks that associate theta-band oscillations with prediction-error generation and feedforward message passing while alpha-band oscillations serve top-down mediated feedback onto lower sensory regions^12,20^.

It is important to highlight that GC estimation in MEG-data is not without limitations. In the current study, we attempted to minimize confounding factors, such as linear mixing, by computing a conditional non-parametric variant of GC in source space that distinguishes direct from indirect interactions. Moreover, we based our inference testing on the difference between two conditions with similar noise levels, volume conduction and interchangeable trial-to-trial variance, which ensures that reported effects were mainly driven by the predictable or mismatch condition of sound sequences. While we cannot exclude the potential confounding effect of evoked activity in the computation of GC in each condition, we addressed this potential issue by applying GC onto short data segments that approximate stationarity^20,64,65^. Moreover, we assessed the robustness of reported effects using trial re-shuffled and time-reversed data, showing that our GC findings could not be accounted for by evoked components or linear noise mixing across regions.

Unlike prior studies^35^ we did not observe changes in GC between HP and vmPFC when comparing mismatch vs predictable sequences. Specifically, Garrido and colleagues^35^ examined visual mismatch processing and modelled phase coupling between vmPFC and HP. The best model involved vmPFC driving HP in the theta-band indicating the updating of online predictions maintained in prefrontal cortices during error detection^62,66^. Prior animal studies have shown a predominant flow in the opposite direction, from HP to PFC^67,68^, supporting a role of the HP in match-mismatch comparisons^69^. Further studies should examine whether connectivity differences between prior studies and our current findings could be accounted for by distinct modelling procedures or differences in the experimental design.

In conclusion, our findings provide novel insights into the contribution of neural oscillations towards auditory predictive processing. Specifically, our data show distinct roles of theta- and alpha-band oscillation in auditory and HP-PFC networks, highlighting that prediction-error generation and message-passing during the detection of mismatch vs. predictable auditory information is mediated through inter-areal rhythmic synchronization. Our results highlight the fundamental role of HP-PFC in this process and suggest that this circuit sustains a domain-general mechanism involved in the representation of sequential information and match-mismatch detection.

## Materials and Methods

### Subjects

Seventeen healthy right-handed human participants took part in the experiment (mean age: 24.9; std: 3.7; range: 20–30 years; 6 males) and were screened for a history of neurological or psychiatric disorders. All participants showed normal hearing levels as assessed with a 5-tone audiometry (250, 500, 1000, 3000, 8000 Hz). Participants were recruited from the University of Glasgow School of Psychology participant pool and provided informed consent prior to the experiment. The experimental protocol was approved by the University of Glasgow College of Science and Engineering Ethics Committee. All methods were performed in accordance with the relevant guidelines and regulations provided by the Code of Ethics of the World Medical Association (Declaration of Helsinki). No subjects were discarded due to excessive head movement (>0.7 cm) or inappropriate hearing levels (mean thresholds ranged from 5–10 dB HL).

### Experimental Design

The experimental paradigm (Fig. 1) involved the consecutive presentation of four-sound series that repeated or differed (p = 0.5; randomly distributed within each block) from a previously presented pattern in the order of the last two sounds. Therefore, sounds in third and fourth positions of the sequence (post-violation interval: 1.2 to 2.1 s) elicited a predictable or mismatch response based on the sequential order of a pre-established auditory template. Mismatch responses were thus contingent on a prior matching process, as defined by the first two sound being identical to those in the preceding sequence, rather than responses to environmental novelty per se. Sounds were composed of sequences of four 300 ms sine-waves separated by a 300 ms fixed inter-stimulus-interval (ISI) and were presented at 600, 1200, and 1800 ms after the onset of the first sound in the sequence. Sound frequencies were randomly selected from a sample of 15 frequencies (100–800 Hz, 50 Hz steps). Each condition-trial was composed of two consecutive quartets separated by 1 s fixed inter-quartet-interval. The first quartet (Q1) consisted of a sequence of sounds and the second quartet (Q2) presented the same four sounds in two conditions: (1) an exact repetition of the previous sequence (“Predictable”) or (2) the same sequence with a mismatch in the order of the last two sounds (“Mismatch”). Condition-trials were separated by 1 s fixed intervals, identical to inter-quartet-intervals, thus making the trial-structure (Q1 followed by Q2) not explicit to the participants. All sequences presented throughout the experimental presentation were unique and were repeated only once during Q2 in the predictable condition. Only data from Q2 quartets containing the experimental manipulation were used in subsequent analyses. Data from Q1 quartets that defined the sensory template were only used for the computation of the covariance matrices. Unlike previous studies^34–36^, we did not include a shuffled or unpredictable condition, hence focussing on associative or prediction-based comparisons rather than novelty per se.

Participants were engaged in an orthogonal auditory 1-back task where a repetition of the same tone within the quartet had to be detected via a button-press with the right-index finger. Twenty target trials were randomly interspersed and presented in quartets (probability = 0.2) that were not followed by a Q2, thus disrupting the predominant Q1–Q2 trials structure (probability = 0.8). Participants performed at a high level (hit rate = 0.87, SD = 0.1). Target trials were excluded from further analysis. A white fixation cross was presented in the middle of the screen and provided target-related feedback by changing its colour to green for detected targets or red for missed and false alarm responses. All stimuli were created using Matlab at a sampling rate of 44.1 kHz and 16-bit resolution. Sounds were delivered binaurally through 6-meter tubes attached to earplugs (ER-30 system, Etymotic Research Inc., IL, USA) inserted into the ear canal and presented at a comfortable listening level adjusted by each participant (60–70 dB SPL). The experiment was implemented using the Psychophysics Toolbox^70^ and was presented in 3–5 blocks, each containing 40 trials per condition and lasting 9.5 min.

### Data Acquisition and Pre-processing

MEG data were acquired using a 248-magnetometer, whole head MEG system (MAGNES® 3600WH, 4D-Neuroimaging, CA, USA). Head position was assessed before and after each acquisition run via five coils co-digitized with participants’ head shape (FASTRAK®, Polhemus Inc., VT, USA) for subsequent co-registration with individual magnetic resonance imaging (MRI; 1 mm^3^; T1-weighted 3D-MPRAGE).

Sensor-level pre-processing was performed using the Fieldtrip Toolbox^71^. Raw MEG signals were 0.5 Hz high-pass filtered (FIR filter, order 2) and 50 Hz power-line noise was removed using a sharp discrete Fourier transform filter. Signals recorded by the MEG reference sensors were used to reduce environmental noise using FieldTrip’s ft_denoise_pca function. Continuous data were down-sampled to 508.6 Hz and epoched in trials of 4.1 s length (1 s pre-stimulus) time-locked to the onset of the first sound in the sequence. Five excessively noisy or flat sensors were discarded from all analyses. Trials contaminated by squid jumps and amplitude ranges above ±7pT were removed prior to independent component analysis decomposition. Independent components containing blinks, eye movements, and cardiac activity were projected out from the data. Resulting signals were visually inspected and trials containing artefacts were manually removed. Discarded sensors were replaced using a triangulation method but were not used for source estimation. An average (±std) of 150.4 trials (±17.8) in the mismatch Q2 condition and 149.6 trials (±16.5) in the predictable Q2 condition survived artefact rejection.

### Whole-Brain MEG-Analysis

Individual MRIs were co-registered to the MEG coordinate system using participants’ landmark information and digitised head shape. Grey and white matter was automatically segmented using Freesurfer^72,73^. The Brainstorm toolbox^74^ was used to create whole-brain volumes using an overlapping spheres method^75^. A template source space (5 mm spacing, 10137 grid points) was defined for each individual volume. The noise covariance matrix used to calculate the inverse operators was estimated from baseline intervals preceding the onset of the first sound in each series (−500 to −3 ms), as obtained from data across all trials. Trial-level source activity was computed using dSPM^76^. For whole-brain dSPM maps, trials in each condition were first averaged and then band-pass filtered in the theta-band (finite impulse response, range: 4–8 Hz, filter order: 1538). The norm of each orientation per voxel was calculated to facilitate visualization and statistical comparison. Individual maps were projected to a template brain and a Gaussian smoothing kernel of 3 mm FWHM (full-width half-maximum) was applied prior to statistical analysis. Individual auditory mismatch effects were obtained by computing dSPM maps on the difference between mismatch and predictable averaged trials, rather than for each condition separately. Therefore, individual dSPM maps do not provide information about the direction of the effects but instead show a modulation induced by the experimental manipulation. The percentage of all grid points in the left and right HP that exceed the 75% of the maximal amplitude was calculated for each individual.

### Region of Interest Definition

Regions of interest (ROIs) data were derived from cortically-constrained source models. Following the same procedure described above, cortical and hippocampal structures obtained from Freesurfer’s automatic subcortical segmentation^77,78^ were combined to compute cortically-constrained source space models (~5 mm spacing, ~7500 dipole locations per hemisphere) using the Boundary Element Method. Such brain models constrain the source space onto the individually-defined cortical mantle^76^, thus applying accurate anatomical and electrophysiological constraints in terms of dipole location, orientation, and current density^57,59^. Based on current whole-brain results and literature findings^32,35,41,42,79^ reporting the involvement of selected regions in match and mismatch detection, we obtained source time-series by averaging all vertex-signals in the following ROIs from the Desikan-Killiany atlas^80^: transverse temporal or Heschl’s gyri (HG; 44–28 vertices, area = 4.93–3.39 cm^2^), the superior temporal gyri (STG; 297–296 vertices, area = 36.05–35.29 cm^2^), rostral middle or frontal dorsolateral prefrontal cortices (dlPFC; 421–426 vertices, area = 38.41–39.98 cm^2^), and lateral and medial orbitofrontal cortices overlapping ventromedial prefrontal cortices (vmPFC; 355–380 vertices, area = 41.13–42.33 cm^2^). The HP ROIs (745–725 vertices, area = 23.06–23.65 cm2) were defined using all the grid points in HP structures that were extracted using FreeSurfer’s subcortical segmentation^77^. Subsequent time-frequency and connectivity analyses were carried out using depth-weighted MNE^81,82^ trials extracted from each ROI. To compute evoked responses noise-normalized dSPM trials were extracted from each ROI, band-pass filtered (finite impulse response, range: 1–30 Hz, filter order: 1538), and averaged per condition.

### Time-Frequency Analyses

Time-frequency representations (TFRs) were calculated for each ROI (−1 to 3.1 s, from sequence onset) for frequencies up to 40 Hz using a Hanning taper method with a 500 ms fixed sliding window length (0.5 Hz steps using the mtmconvol function), centred every 10 ms. Initial and final 250 ms segments were clipped from TFRs to exclude edge effects. Total power was expressed as relative change (750 ms baseline interval, prior to sequence onset). Phase-locking or Inter-trial phase coherence (ITPC) was calculated to assess the consistency of brain responses over trials using the same parameters as for total evoked power (using complex Fourier-spectra). Phase-locking corresponds to the magnitude of the amplitude-normalized complex numbers averaged across trials of the TFR estimates for each time-frequency bin^83^. Phase-locking values range from 0 to 1, reflecting zero to near-perfect phase consistency across trials.

### Spectral Granger Causality Analysis

We examined the causal influence between ROIs (HG, STG, HP, vmPFC, dlPFC) using Granger Causality (GC)^84^. GC spectrum was obtained in a non-parametric manner by computing Geweke’s frequency domain version of conditional GC^85,86^. As opposed to a pairwise approach, conditional GC takes into account information from all ROIs when estimating the interaction terms, thus distinguishing direct from potentially indirect effects caused by a third source. In order to reduce non-stationarity, trial-condition data during the post-violation interval was de-trended and segmented into 3 non-overlapping 300 ms intervals. Spectral density matrices for each segment were obtained from the Fourier decomposition, calculated from 0 to 40 Hz using a single 500 ms sliding window (0.5 Hz spectral resolution, 10 ms temporal resolution) on ±6 s padded data. Spectral matrices were factorized and combined with Geweke’s time series decomposition to estimate conditional GC. GC values were then averaged across segments prior to statistical testing. The rationale behind data segmentation is that shorter segments are less likely to show non-stationarity^64,65^, and the inclusion of larger data-points results in smoother shapes of the cross-spectral densities and more stable results for the non-parametric formulation of GC^20,87,88^.

Confounding factors such as linear noise mixing due to volume conduction and noise correlation across regions may lead to spurious and false positive GC results. To address this issue, we compared our original results with GC-estimates obtained using time-reversed data^89,90^. In addition we assessed the potentially adverse effect caused by trial-to-trial variability of evoked components, leading to low-stationary time-series^88,91^. We computed GC on surrogate trial-shuffled data and compared original and surrogate results, on the basis that causality would be lost during reshuffling leaving only the contribution of non-stationary evoked components.

### Statistical Analyses

Group-level statistical testing of whole-brain evoked theta activity (n = 17 subjects) employed a Monte Carlo method with cluster-based maximum correction for multiple comparisons^92^. Dependent-samples t-tests were used to identify clusters of contiguous data points showing a difference between conditions (Mismatch vs Predictable), thresholded at p < 0.05, two-tailed. The sum of *t* statistics over all data points was used to calculate cluster size. Effect sizes for cluster-based t-statistics are reported as the summed t-values across all bins within a cluster (T_sum_). Time-bins within the post-violation interval were averaged prior to permutation testing. The null distribution was estimated using 1000 permutations and effects were clustered based on spatial adjacency (cluster-corrected at p < 0.005).

For group-level TFR maps, condition differences within each ROI were independently assessed between 1.2 and 2.9 s and from 0.5 to 40 Hz using 2000 permutations. Effects were clustered in both time and frequency dimensions (cluster-corrected at p < 0.05).

Participants-averaged evoked responses within each ROI were initially compared across conditions using non-parametric two-sample t-tests (3000 permutations, FDR corrected across ROIs at p < 0.05, minimum threshold of 7 consecutive time-bins = 10–12 ms). T- and p-values across consecutive significant time-bins are reported. In addition, condition effects were tested during the specific time-intervals of the MMN and the P300-response. Based on grand-averaged data, mean activity was extracted from the two MMN (40 ms interval around peak) and P300 components (80 ms interval around peak), peaking respectively at ~120 ms and ~220 ms after the onset of the third and fourth sounds in the quartet. Mean activity across conditions in each ROI (n = 10) and component (n = 4) was statistically tested using non-parametric permutation-based testing (10000 permutations, FDR corrected across ROIs at p < 0.05).

Condition differences in original GC spectrograms and surrogate datasets were assessed independently in each hemisphere within the post-violation interval and for all frequencies. The null distribution was estimated using 1000 permutations and effects were clustered in the frequency dimension (cluster-corrected at p < 0.05).

## Data Availability

The datasets generated during and/or analysed during the current study are available from the corresponding author on reasonable request.
